# A putative amino acid transporter determines sensitivity to the two‐peptide bacteriocin plantaricin JK

**DOI:** 10.1002/mbo3.363

**Published:** 2016-05-05

**Authors:** Camilla Oppegård, Morten Kjos, Jan‐Willem Veening, Jon Nissen‐Meyer, Tom Kristensen

**Affiliations:** ^1^ Biochemistry and Molecular Biology SectionDepartment of BiosciencesUniversity of OsloP.O. Box 1066 BlindernOslo0316Norway; ^2^ Molecular Genetics GroupGroningen Biomolecular Sciences and Biotechnology InstituteCentre for Synthetic BiologyUniversity of GroningenNijenborgh 7 9747 AGGroningenThe Netherlands

**Keywords:** Antibacterial activity, bacteriocins, membrane proteins, mode of action

## Abstract

*Lactobacillus plantarum* produces a number of antimicrobial peptides (bacteriocins) that mostly target closely related bacteria. Although bacteriocins are important for the ecology of these bacteria, very little is known about how the peptides target sensitive cells. In this work, a putative membrane protein receptor of the two‐peptide bacteriocin plantaricin JK was identified by comparing Illumina sequence reads from plantaricin JK‐resistant mutants to a crude assembly of the sensitive wild‐type *Weissella viridescens* genome using the polymorphism discovery tool VAAL. Ten resistant mutants harbored altogether seven independent mutations in a gene encoding an APC superfamily protein with 12 transmembrane helices. The APC superfamily transporter thus is likely to serve as a target for plantaricin JK on sensitive cells.

## Introduction

Many species of lactic acid bacteria produce nonmodified, ribosomally synthesized antibacterial peptides (bacteriocins) that kill target bacteria by membrane permeabilization (Nissen‐Meyer et al. 2009; Cotter et al. 2013). In many cases, the bacteriocins do not act by directly perturbing the membrane lipids, but rather by binding to a specific membrane protein (the “bacteriocin receptor”), where the interaction between peptide and receptor protein leads to membrane leakage and cell death (Ramnath et al. 2000; Héchard and Sahl 2002; Diep et al. 2007; Uzelac et al. 2013; Kjos et al. 2014). Although the events that lead to leakage are not well characterized, there are indications that the mechanism involves conformational changes in the receptor protein upon binding of the bacteriocin (Diep et al. 2007; Kjos et al. 2010a). This receptor‐mediated mechanism may explain both the extreme potency of many bacteriocins (antimicrobial activity in the pico‐ to nanomolar range) and their often narrow inhibitory spectra. Also involved in the mode of action of bacteriocins are the immunity proteins that bacteriocin‐producing cells synthesize to protect themselves against the action of their own bacteriocins. In the receptor‐mediated model of bacteriocin action, the immunity protein acts by interacting with the bacteriocin‐bound receptor and thereby prevents membrane leakage (Diep et al. 2007).

Bacteriocins are of interest both due to their importance for interactions between bacterial species in natural habitats, and as alternatives to classical antibacterials to combat pathogens and food spoilage bacteria. However, we still lack knowledge about the nature of the interactions between bacteriocins, receptors, and immunity proteins, and in many cases we do not even know the identities of these three components. Generally, the immunity protein is encoded in the same operon as the bacteriocin (van Belkum et al. 1991; Cotter et al. 2013) and thus easy to identify. The identification of the receptor protein of a given bacteriocin is less trivial, and the receptors of many bacteriocins are consequently still unknown. The IIC and IID subunits of the mannose phosphotransferase system were, by a combination of genetic and biochemical analyses, shown to act as receptors for the pediocin‐like (class IIa) bacteriocins (Ramnath et al. 2000; Gravesen et al. 2002; Héchard and Sahl 2002; Diep et al. 2007). The same transport system also functions as a receptor for lactococcin A and microcin E492, bacteriocins that structurally are quite different from each other and from the pediocin‐like bacteriocins (Bieler et al. 2006; Diep et al. 2007). A glucose‐specific PTS system is also involved in sensitivity to the glycosylated bacteriocin sublancin. However, in this case the mode of action does not seem to involve compromised membrane integrity or pore formation (Garcia De Gonzalo et al. 2015). Other identified bacteriocin receptor proteins are the maltose ABC transporter (garvicin ML, a class IIc bacteriocin) (Gabrielsen et al. 2012) and the metallopeptidase YvjB (the class IId bacteriocin LsbB) (Uzelac et al. 2013).

Recently we identified the receptor protein of lactococcin G, a two‐peptide bacteriocin belonging to class IIb (Kjos et al. 2014). Bacteriocins in this class consist of two different peptides that must be present in equimolar amounts for full antibacterial activity. By whole‐genome sequencing of lactococcin G‐resistant mutants of a sensitive strain, we could identify the lactococcin G receptor as the membrane‐embedded enzyme UppP, an undecaprenyl pyrophosphate phosphatase that is involved in cell‐wall synthesis. When compared with the annotated Genbank genome of the target strain, the resistant mutants all had point mutations in or close to the *uppP* gene leading to production of a truncated protein or to reduced protein expression. In the present work, we have used a similar approach to identify a protein involved in the mode of action of plantaricin JK, a two‐peptide bacteriocin produced by strains of *Lactobacillus plantarum* (Anderssen et al. 1998). Like lactococcin G, plantaricin JK is most active when the two peptides are present in equimolar amounts, although the peptides also show a much reduced activity on their own. Plantaricin JK is encoded in the plantaricin locus of *L. plantarum* by the genes *plnJ* and *plnK*, which are cotranscribed with the immunity genes *plnLR* (Diep et al. 1995, 2009; Kjos, Snipen, et al. 2010b). The two genes encode peptides of 25 (PlnJ) and 32 (PlnK) amino acids that act together to permeabilize the membrane of susceptible cells, resulting in a drop in electric potential and pH gradient, and eventually cell death (Moll et al. 1999). The peptides form amphiphilic alpha‐helices and interact via so‐called GxxxG motifs (Hauge et al. 1999; Rogne et al. 2009). Plantaricin JK is known to only target strains that are closely related to the producer (Anderssen et al. 1998), but the underlying reasons for this have remained unknown. Here, we shed light on the mode of action of plantaricin JK, information that will be important for the further understanding of how plantaricin genes contribute to the population ecology of lactobacilli (Riley and Wertz 2002).

## Experimental Procedures

### Production and purification of plantaricin JK

The PlnJ and PlnK peptides of plantaricin JK were obtained by solid‐phase synthesis as previously described (Hauge et al. 1999). Synthetic plantaricin EF peptides were obtained from Genscript. The concentrations of the peptides in solution were estimated by measuring the absorbance at 280 nm and using an absorbance coefficient calculated from the content of aromatic amino acids.

### Bacteriocin activity assays

The antimicrobial activity of plantaricin JK and plantaricin EF against sensitive and resistant target cells was tested using a 96‐well microtiter plate‐based activity assay similarly as previously described (Oppegård et al. 2007, 2008, 2010). When measuring the antimicrobial activity of plantaricin JK and EF against wild‐type and mutant *Weissella viridescens*, overnight (stationary phase) cultures of these cells were diluted about 1:50 in MRS medium, and 200 *μ*L of this cell suspension was added to each well together with twofold dilutions of the bacteriocin. The microtiter plates were then incubated for 6–8 h at 30°C before the growth inhibition was measured spectrophotometrically at 600 nm. The minimum inhibitory concentration (MIC) is defined as the peptide concentration (the sum of both peptides [in a 1: 1 ratio]) that inhibited growth by 50%.

### Generation of plantaricin JK‐resistant mutants

The plantaricin JK‐sensitive strain *W. viridescens* NCDO 1655 (MIC about 0.5 nmol/L) was used as a source of spontaneous mutants with increased resistance to the bacteriocin. This strain was used since it is also sensitive to plantaricin EF. The strain was routinely cultured in MRS media without additions. A culture of *W. viridescens* from a single colony was diluted about 1:100 with MRS media containing twofold dilutions of plantaricin JK (at concentrations ranging from about 1–20 nmol/L). The cells were cultured about 24 h at 30°C in 6‐mm wells of a 96‐well microtiter plate. Cultures from wells in which cell growth was obtained were diluted 1:10 with MRS medium and stored at either 30°C or 4°C for about 100 h, and then diluted about 1:100 with MRS media containing twofold dilutions of plantaricin JK (concentrations ranging from about 5–50 nmol/L). The cells were then cultured between 70 to 100 h at 30°C in 6‐mm wells of a 96‐well microtiter plate. Cells from wells in which cell growth was obtained were plated on MRS agar plates and individual colonies were picked. Cells from each colony were tested for resistance to plantaricin JK using the bacteriocin activity assay. This resulted in 10 plantaricin JK‐resistant strains (JK1‐6, JK8‐11). To assess the stability of the resistance phenotype, the resistant cells were grown for several generations in MRS medium without added bacteriocin, and then again tested for resistance. We observed no decrease in resistance after prolonged growth in the absence of bacteriocin, indicating that resistance was due to mutation rather than to adaptation.

### Isolation of genomic DNA and whole‐genome sequencing

DNA was isolated from 1.5 mL overnight cultures of the sensitive wild‐type strain and the 10 resistant mutants using the Qiagen Blood and Tissue Kit (Qiagen NV, The Netherlands) according to the producer's recommendations. DNA samples were submitted to The Norwegian Sequencing Centre (sequencing.uio.no). The samples were sequenced by 16× multiplexing in an Illumina MiSeq instrument, giving 1.5–2.5 million 250 nt long paired‐end reads per strain.

### Assembly of the reference genome

Since no reference genome of *W. viridescens* NCDO 1655 is available in the public databases, we made assemblies of the wild‐type paired‐end sequence reads (in some instances after merging overlapping paired‐end reads with FLASH (Magoc and Salzberg 2011)) using publicly available software for de novo assembly (ABySS (Simpson et al. 2009), SPAdes (Bankevich et al. 2012), Velvet (Zerbino and Birney 2008), and the assembly program in Geneious (Geneious version 7 created by Biomatters, available from http://www.geneious.com)). The raw assemblies were then used as reference genomes in the polymorphism detection software VAAL (Nusbaum et al. 2008).

### Comparison of Illumina reads from the mutated strains with the assembled reference genome

Illumina reads were compared with the wild‐type genome contigs using the polymorphism discovery tool VAAL.

Contigs harboring differences reported by VAAL were annotated for probable gene content using a combination of Glimmer 3 and Blast searches. VAAL hits were evaluated and verified by aligning relevant reads to individual contigs using the Align/Assemble tool and the Find Variations/SNPs tool in the Geneious suite. This showed that all mutations detected by VAAL were located in position with a high coverage of mutant reads (154–484×), that both strands were equally represented in the reads, and that the mutations were present in 90–100% of the reads (detailed results not shown).

## Results

### Increased plantaricin JK resistance of mutated *W. viridescens* strains

Strains that are sensitive to plantaricin JK include *L. plantarum*,* L. sakei,* and *W. viridescens* (previously known as *L. viridescens* (Collins et al. 1993)), all close relatives of the producer. Sensitive *W. viridescens* NCDO 1665 was exposed to increasing concentrations of plantaricin JK in liquid culture prior to plating. Using this method, we collected 10 *W. viridescens* colonies with increased resistance against plantaricin JK. As shown in Table 1, the MIC values for the strains derived from the 10 isolated colonies were 100–600 times higher than for the parental strain (0.5 nmol/L). Genomic DNA was isolated from each of the 10 strains with increased resistance, as well as the parental strain, and sequenced.

**Table 1:** Increased resistance of *W. viridescens* mutants toward plantaricin JK and plantaricin EF. The mutants were isolated and minimum inhibitory concentration (MIC) values determined as detailed in the Experimental procedures section

Mutant strain	Fold increase of MIC value	
	Plantaricin JK	Plantaricin EF
JK1	100 ± 20	1
JK2	300 ± 100	1
JK3	300 ± 100	1
JK4	600 ± 200	2 ± 1
JK5	600 ± 200	1
JK6	600 ± 200	1
JK8	300 ± 100	1
JK9	300 ± 100	1
JK10	100 ± 50	1
JK11	400 ± 100	7 ± 3

Strains that produce plantaricin JK, for example, *L. plantarum* C11, also produce another two‐peptide bacteriocin, plantaricin EF (Anderssen et al. 1998; Diep et al. 2009). *W. viridescens* NCDO 1655 is even more sensitive to this bacteriocin than to plantaricin JK (MIC = 0.05 nmol/L). To investigate the specificity of the mutants, we also tested how the plantaricin EF sensitivity was affected. As shown in Table 1, there was no significant reduction in the sensitivity of the mutants, compared to the wild‐type strain, except for mutant JK11, which was marginally more resistant to plantaricin EF than the wild‐type. This indicates that the increased resistance of the mutants to plantaricin JK is not a general effect on the bacteriocin resistance, but an effect that is specific for plantaricin JK.

### Assembly of a reference genome

As described in the Experimental procedures section, we made several assemblies of the wild‐type genome, using a selection of assembly tools. The Geneious assembly program used on FLASH‐merged reads gave the lowest number of contigs (243), and this assembly was the only one that gave a reasonably low number of polymorphic sites in the subsequent VAAL analysis. Thus, this 243‐contig assembly was used for further analyses. The contigs added up to a total of 1.59 Mbp. A 34 contig assembly of the genome of a different *W. viridescens* strain, DMS 20410, was recently added to the NCBI database (NZ_JQBM01000000, published 12‐11‐2015). The genome representation is described as full, and the genome size is given as 1,537,173 bp. Assuming that the genome sizes of the two strains are similar, this would indicate that the 243 contigs represent close to the full genome.

### Localization of mutations

Whole‐genome sequence reads from the 10 highly plantaricin JK‐resistant colonies as well as wild‐type reads were compared to the reference genome using VAAL. In total, 58 differences between the sequence reads from the analyzed strains and the reference genome contigs were detected (Table S1). Of these, 11 differences were present in the bacteriocin‐sensitive wild‐type, as well as in several or all of the resistant strains. All these differences were localized to regions of the assembly with a very low coverage (1–5× rather than the average 50×), that is, regions in the assembly where errors in the consensus sequence are most likely to occur. Consequently, these 11 differences were regarded as assembly errors and were not considered in the further analysis. Likewise, another 11 differences reported by VAAL as present in several of the mutant genomes were localized to low‐coverage regions with unreliable consensus sequences; these were also excluded from further consideration (the excluded differences are labeled in red in Table S1). The remaining 36 differences reported by VAAL between resistant and wild‐type genomes are given in Table 2.

**Table 2:** Differences in genome sequence between the sensitive wild‐type and resistant mutants of *W. viridescens*. The Table is based on Supplementary Table 1, but false positives have been removed as detailed in the Methods section

Contig no.	Position in contig	Coverage in assembly	Genetic location of difference	Mutant strains										
				VT1 (wild‐type)	JK1	JK2	JK3	JK4	JK5	JK6	JK8	JK9	JK10	JK11
Contig 3	15,305	5	Silent mutation in D‐Ala‐D‐Ala carboxypeptidase								X			
Contig 7	17,428	16	ACG‐ATG = 40T‐M in S4 RNA‐binding domain protein					X	X					
Contig 10	2518	44	CGA‐TGA = 104R‐stop in APC family amino acid‐polyamine‐organocation transporter, 610 aa				X							
Contig 10	2739	30	TTC‐TTTC = 177F‐frameshift in APC family amino acid‐polyamine‐organocation transporter					X	X	X				
Contig 10	3337	18	GCG‐ACG = 377A‐T in APC family amino acid‐polyamine‐organocation transporter			X								
Contig 10	3367	19	TCT‐CCT = 387S‐P in APC family amino acid‐polyamine‐organocation transporter		X									
Contig 10	3393	23	TGG‐TGA = 395W‐stop in APC family amino acid‐polyamine‐organocation transporter										X	
Contig 10	3482	21	ATG‐AAG = 425M‐K in APC family amino acid‐polyamine‐organocation transporter											X
Contig 10	3491	22	CAT‐CGT = 428H‐R in APC family amino acid‐polyamine‐organocation transporter								X	X		
Contig 10	12,129	6	GGT‐AGT = G‐S in nucleic acid‐binding protein									X		
Contig 11	2629	6	GTA‐ATA = V‐I in predicted metal‐dependent hydrolase						X					
Contig 12	3366	15	GTG‐GCG = V‐A in Met‐tRNA formyl transferase			X								
Contig 15	5486	18	Intergenic region according to Glimmer, no BlastX hits										X	
Contig 17	10,149	45	Just downstream of Glimmer orf 10 branched chain amino acid aminotransferase		X									
Contig 23	2907	10	ATT‐GTT = I‐V in UTP–glucose‐1‐P uridylyltransferase					X						
Contig 23	13,717	32	GTG‐GGTG = frameshift in glutamine ABC transporter, permease/substrate‐binding protein								X	X		
Contig 26	9532	60	ATT‐GTT = I‐V in purH, bifunctional phosphoribosylaminoimidazolecarboxamide formyltransferase/IMP cyclohydrolase								X	X		
Contig 28	11,172	14	GGA‐GGGA = frameshift 55 aa from end of integral membrane protein		X									
Contig 36	7084	57	GCT‐GTT = A‐V in oxoacyl‐ACP synthase			X								
Contig 37	167		GAA‐GAG = E‐E silent mutation in aspartate kinase			X								
Contig 42	4874	6	CTA‐CCA = L‐P in putative dienelactone hydrolase (no gene predicted by Glimmer)					X	X	X				
Contig 47	2473	7	Deletion of T in Intergenic region according to Glimmer											
Contig 64	3734	4	AGT‐AGC = silent mutation in nucleotide‐binding protein					X						
Contig 69	1283	14	GGT‐AGT = G‐S in gene with similarity to HTH AraC regulatory protein		X									
Contig 73	2467	19	Frameshift after amino acid 187 in PTS system mannose family transporter subunit IID protein.		X									
Contig 77	233	28	CAC‐CAT = Silent mutation in pyruvate carboxylase					X	X	X				
Contig 79	2182	15	GAC‐AAC = D‐N in putative uncharacterized protein. Verified to be a mutation		X									
Contig 84	4263	8	BLAST and Glimmer: not coding region, downstream of GTP‐binding protein TypA gene											X
Contig 85	8117	5	AAA‐AAAA = frameshift toward end of Glimmer prediction, no Blast similarity to anything											X
Contig 103	928	81	ATT‐ATC = silent mutation in glutathione reductase										X	
Contig 111	1948	30	AAT‐AAC = silent mutation in oxidoreductase						X					
Contig 114	6645	2	GGA‐GAA = G‐E in MccC family protein – putative peptidase							X				
Contig 132	1556	6	Intergenic region											X
Contig 146	971	23	CAG‐CGG = Q‐R in acetylornithine deacetylase						X					
Contig 153	1131	2	6 nt downstream of penicillin‐binding protein/beta‐lactamase			X								
Contig 160	2010	5	6 nt downstream of conserved hypothetical protein, putative receptor										X	

**Figure 1:**
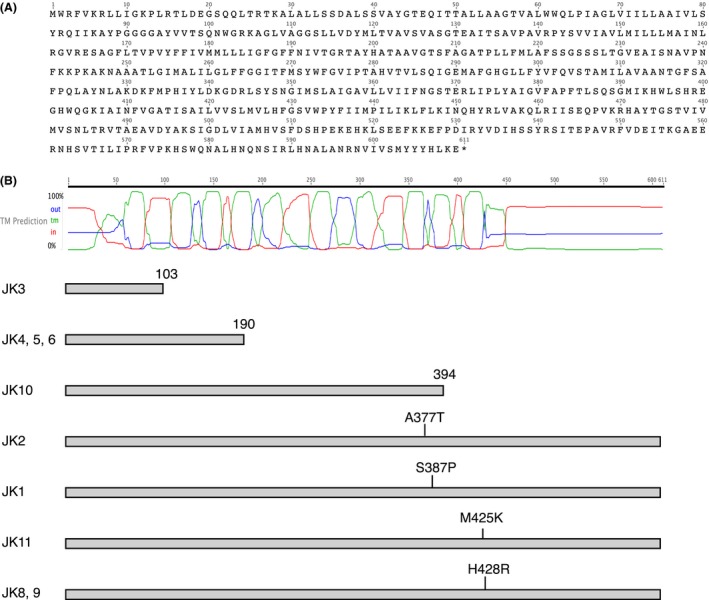
The APC superfamily protein encoded by the gene that was mutated in the 10 plantaricin JK‐resistant strains. (A) The amino acid sequence of the protein. (B) The predicted membrane topology of the protein. The green curve shows predicted transmembrane regions, the red curve cytoplasmic regions, and the blue curve extracellular regions. Below these curves, the protein products expected from the gene mutations are shown schematically for the 10 mutants.

Remarkably, all 10 plantaricin JK‐resistant strains harbored mutations in a region in contig 10 between positions 2518 and 3491. Contig 10 is 31 kb long, and the coverage in this part of the contig was 13–45 fold. The gene prediction software Glimmer 3 (Delcher et al. 2007) predicted a gene in contig 10 from position 2209 to 4041. A comparison of the encoded amino acid sequence (610 aa) with the Genbank nonredundant protein database using Blast revealed that the predicted gene encoded a protein with 87–88% similarity to proteins from various *Weissella*,* Leuconostoc,* and *Lactobacillus* species. These proteins are annotated as APC family amino acid‐polyamine‐organocation transporters, amino acid permeases, and amino acid transporters. A comparison of the sequence to the Pfam database of protein domains revealed the presence of a Pfam family AA_permease_2 domain (PF13520). The amino acid sequence of the protein, and the positions of predicted transmembrane helices (predicted using Geneious) are shown in Fig. 1, along with the positions of the mutations in the resistant isolates.

The transmembrane (TM) prediction tool in the Geneious suite predicted 12 TM helices in the encoded protein (Fig. 1A). The predicted positions of the 12 TM helices correspond to their positions in other members of this protein superfamily (Reig et al. 2007). The resistant strains JK4, 5, and 6 contain an identical nucleotide change in the gene and may be results of a single mutational event. The same is the case for JK8 and 9. Three of the identified mutations lead to truncation of the protein: in JK3 after amino acid 103 (between TM helix 2 and 3), in JK4, 5, and 6 after amino acid 190 (in the middle of TM helix 5, due to a frameshift mutation) and in JK10 after amino acid 394 (after TM helix 10). The remaining four independent mutations are single amino acid changes occurring between position 377 and 428 (TM helix 10 and the predicted extracellular region between TM helix 11 and 12).

Nine of the 10 resistant strains contained one or more additional mutations located outside the APC family transporter gene (Table 2). However, these mutations appear to be randomly occurring in different genes, and we could not find any patterns that could explain the resistant phenotype. Importantly, in one strain (JK3 with 300‐fold increased resistance), the mutation in the APC superfamily gene was the only difference to the wild‐type genome that was detected.

We have made several efforts to verify the essential role of the APC family protein in the function of plantaricin JK, both by reintroducing the wild‐type gene in resistant *W. viridescens* mutants and by introducing the gene in resistant *Lactobacillus* strains using different cloning strategies. However, we did not succeed in obtaining viable cells carrying the intact transgene of this large membrane protein.

## Discussion

### Identification of mutations leading to resistance toward plantaricin JK

In our previous identification of the receptor of the two‐peptide bacteriocin lactococcin G (Kjos et al. 2014), we identified mutations in the sensitive target strains *L. lactis* IL1403 and MG1363, for which extensively annotated, fully assembled genomes were available in the sequence databases. In the present work, no reference genome was available for any plantaricin JK‐sensitive strains. As an alternative we used a crude assembly of the *W. viridescens* genome. Typically, crude assemblies of microbial genomes by short reads will consist of a large number of contigs (in our case 243) that cannot easily be assembled further into a single genome, due to errors in the assembly process. These errors are mainly due to repetitive regions in the genome (e.g., genes for ribosomal RNA). Assembly software will frequently misplace reads from such regions, leading to deterioration of consensus sequences and consequent problems in the further assembly. When compared with mutant sequence reads, errors in the consensus sequences may erroneously be reported as polymorphisms. We found that most of such false positives could be removed by including a comparison of the wild‐type sequence reads and the assembly constructed from them in the polymorphism analysis, and by disregarding differences reported in low‐coverage regions of the assembly. Long‐read sequencing such as Pacbio SMRT sequencing might provide a better alternative for contig assembly but is at the moment still significantly more expensive than short read Illumina sequencing.

### The APC family protein is a likely plantaricin JK receptor

The results from the sequence comparisons revealed a clear correlation between plantaricin JK resistance and mutations in a contig 10 gene encoding an APC family protein, and thus strongly suggest that the APC family protein is involved in the mode of action of the bacteriocin that eventually leads to membrane leakage (Moll et al. 1999). The most likely role of the APC protein would be as a receptor protein. The APC protein has several of the properties expected for a bacteriocin receptor: similar to other identified bacteriocin receptors (Diep et al. 2007; Gabrielsen et al. 2012; Uzelac et al. 2013; Kjos et al. 2014) it is a transmembrane protein, with regions of the polypeptide chain exposed on the outer surface of the membrane. Like several of the other identified bacteriocin receptors (mannose PTS permease, glucose PTS permease, and maltose ABC transporter) it is a transport protein, which means that it possesses some kind of pore‐opening mechanism that could be hijacked and forced to open due to bacteriocin binding. Furthermore, the APC family protein sequence of *W. viridescens* is only 76.4% identical to the closest relative in the public databases and thus considerably different from its homologs in other sequenced *Weissella* and *Lactobacillus* strains. Assuming that plantaricin JK uses this protein as a sequence‐specific target, this may explain the narrow inhibitory spectrum of this bacteriocin, which only targets a small subset of strains within these genera.

Also relevant here is the fact that except for JK11, which was marginally (fivefold) more resistant than the wild‐type, the mutants with increased resistance toward plantaricin JK are as sensitive as the wild‐type toward the two‐peptide bacteriocin plantaricin EF. This indicates that the resistance was specific against plantaricin JK, and that plantaricin JK and EF have different receptors, consistent with an earlier study that showed that plantaricin EF causes leakage of small monovalent cations, but not anions, whereas plantaricin JK causes leakage of some anions, but not cations (Moll et al. 1999).

### Position of the mutations in the APC family protein

The APC superfamily of transporters specific for amino acids, polyamines, and organocations is one of the largest protein superfamilies and has members in all kingdoms of life (Jack et al. 2000; Reig et al. 2007). In the motif database Pfam the APC superfamily has been subdivided into 20 families, and based on sequence comparisons, the putative plantaricin JK receptor should belong to the AA_permease_2 family.

As shown in Figure 1B, three of the seven identified mutations lead to truncation of the APC family protein and removal of two, seven, and ten TM helices, respectively. Most likely, these truncations all result in an inactive protein. Four of the mutations lead to amino acid substitutions. Two of these are present in TM helix 10, which according to the prediction tool MEMSAT‐SVM ((Nugent and Jones 2012), http://bioinf.cs.ucl.ac.uk/psipred/?memsatsvm=1) may be a pore‐lining helix, whereas the remaining two are localized to the extracellular (according to predictions) region between TM helix 11 and 12. The positioning of these mutations to the vicinity of a membrane pore and to an extracellular loop, respectively, is fully consistent with their effect on the action of the bacteriocin.

Generally, there is a good correlation between the nature of the mutations and the degree of resistance they lead to. Thus, two of the truncations (JK3 and JK4, 5, 6) lead to 3–600 fold increased resistance, whereas the point mutations seem to have a somewhat smaller effect. It is, however, noteworthy that the mutation in JK10, leading to truncation after amino acid 394, gives a lower increase in the resistance than the point mutations in position 425 and 428 in JK8, 9, and 11.

Our identification of an APC family protein as a putative membrane protein receptor of plantaricin JK is fully based on the observed accumulation of point mutations in the encoding gene, where the mutations in some cases lead to the introduction of premature stop codons and in other cases to amino acid residue changes in regions that presumably are important for the bacteriocin/protein interaction. We will continue our efforts to produce the protein by heterologous expression or in vitro expression, both to further verify its function as a plantaricin JK receptor and to study the molecular interactions between the protein, the bacteriocin, and the immunity protein.

## Conflict of Interest

None declared.

## Supporting information


**Table S1.** Each row represents a polymorphism reported by VAAL, with the position of the polymorphism detailed in the leftmost column, followed by information about the strain distribution (wild‐type and 10 mutants) of the polymorphism.
