# Supplementary material for: A putative amino acid transporter determines sensitivity to the two‐peptide bacteriocin plantaricin JK
Source: Microbiologyopen. 2016 May 5;5(4):700–8. doi: 10.1002/mbo3.363 (PMC4985602; doi:10.1002/mbo3.363)
Supplement: Supplementary file 1 — Table S1. Each row represents a polymorphism reported by VAAL, with the position of the polymorphism detailed in the leftmost column, followed by information about the strain distribution (wild‐type and 10 mutants) of the polymorphism. [file MBO3-5-700-s001.pdf]

Supplementary Table 1

Each row represents a polymorphism reported by VAAL, with the position of the polymorphism detailed in the leftmost column, followed by information about the strain distribution (wild-type and 10 mutants) of the polymorphism. The rightmost column describes the genomic position of the polymorphism. Rows in the table containing cells labeled in red were considered to be likely false positives, based on the distribution among the strains and sequence coverage of the polymorphic position in the wild-type genome assembly, as detailed in the text. Rows containing cells labeled in green were considered as representing real polymorphisms and are described further in Table 2 in the main text.

VAAL report (contig, position in contig, sequence context)	WT	JK1	JK2	JK3	JK4	JK5	JK6	JK8	JK9	JK10	JK11	Contig No.	Comment
2 15304 left=CAAGATCAAGTGAAGACAAA sample=A ref=G right=CACGCAGCACACCTCCCTAA								X				Contig 3	Silent mutation in D-Ala-D-Ala carboxypeptidase
6 17427 left=AATTCGTTTCGGGAATAAGA sample=T ref=C right=GTCTGTTTTGCGTATCGATA					X	X						Contig 7	ACG-ATG=T-M in S4 RNA-binding domain protein
9 2517 left=TAACCTCGCAAAATTGGGGA sample=T ref=C right=GAAAAGCTGGTTTGGTGGCC				X								Contig 10	CGA-TGA=R-stop in APC family amino acid-polyamine-organocation transporter
9 2738 left=ACCGTTCCAGTTTACTTTTT sample=T ref= right=CATTGTGATGATGTTGCTGT					X	X	X					Contig 10	TTC-TTTC=frameshift in APC family amino acid-polyamine-organocation transporter
9 3336 left=AACGATTGATTCCATTGTAT sample=A ref=G right=CGATTGGCGTTTTCGCTCCT			X									Contig 10	GCG-ACG=A-T in APC family amino acid-polyamine-organocation transporter
9 3366 left=TTTTCGCTCCTTTCACATTG sample=C ref=T right=CTCAATCAGGTATGATTAAG		X										Contig 10	TCT-CCT=S-P in APC family amino acid-polyamine-organocation transporter
9 3392 left=TCAGGTATGATTAAGCATTG sample=A ref=G right=CTTAGTCATCGTGAGGGGCA										X		Contig 10	TGG-TGA=W-stop in APC family amino acid-polyamine-organocation transporter
9 3481 left=AATTTTAGTTGTTTCGTTGA sample=A ref=T right=GGTGTTACATTTTGGCTCAG											X	Contig 10	ATG-AAG=M-K in APC family amino acid-polyamine-organocation transporter
9 3490 left=TGTTTCGTTGATGGTGTTAC sample=G ref=A right=TTTTGGCTCAGTTTGGCCAT								X	X			Contig 10	CAT-CGT=H-R in APC family amino acid-polyamine-organocation transporter
9 12128 left=TTGAACCGATTCATGTGACC sample=A ref=G right=GTTATATTCAAGCTGATCGA									X			Contig 10	GGT-AGT=G-S in nucleic acid-binding protein
10 2628 left=TTGATGGACATTTACACTTG sample=A ref=G right=TACAAAACATTGCGGGCTTT						X						Contig 11	GTA-ATA=V-I in predicted metal-dependent hydrolase
10 17328 left=GTTGAAAACTAAATTTTTTT sample=A ref=T right=AAAAATGACGTAACAGTTCA			X		X			X		X		Contig 11	Probably not real, only two reads in the assembly

VAAL report (contig, position in contig, sequence context)	WT	JK1	JK2	JK3	JK4	JK5	JK6	JK8	JK9	JK10	JK11	Contig No.	Comment
11 3365 left=TAAGGCTTCAGCACAATCAG sample=C ref=T right=GCACAACCATGTTCGAGGTT			X									Contig 12	GTG-GCG=V-A in Met-tRNA formyl transferase
14 5485 left=TTAAAAATAAACGGATGCTT sample=A ref=G right=CCTCAGGGCAATCATCCTTT										X		Contig 15	Intergenig region according to Glimmer, no BlastX hits
14 16166 left=TGCCTATGGCGGGTCACCTT sample= ref=T right=GGACAAGCGCAGCATTTGAA	X	X	X	X	X	X	X	X	X	X	X	Contig 15	No T in the consensus contig sequence
16 10148 left=AGCGTGTGCATCGTTTTTTT sample= ref=T right=AGCTAATCTAGTGGTACATC		X										Contig 17	Just downstream of Glimmer orf 10 branched chain amino acid aminotransferase
22 2906 left=CTATTGTTGATAAGCCAACA sample=G ref=A right=TTCAATTTATTATCGAAGAG					X							Contig 23	ATT-GTT=I-V in UTP--glucose-1-P uridylyltransferase
22 13716 left=AGCCATTATGTTGGTAAGGG sample=G ref= right=TGCATACAACTATAAGGCTA								X	X			Contig 23	GTG-GGTG=frameshift in glutamine ABC transporter, permease/substrate-binding protein
24 13666 left=AAACTATCTCGAAAGATAAT sample=T ref=C right=CCAAATATAATACTTAACGC		X	X		X	X		X		X		Contig 25	Only one read in ref, low quality
25 9531 left=GGATTGGGCAAGGGGACACC sample=G ref=A right=TTGAACAAGCTTGGGATCGC								X	X			Contig 26	ATT-GTT=I-V in purH, bifunctional phosphoribosylaminoimidazolecarboxamide formyltransferase/IMP cyclohydrolase
27 11172 left=AATTCCTGGTGGGGCGGGGG sample= ref=G right=AGCAGAGATTGGTTTCTCAG		X										Contig 28	GGA-GGGA=frameshift 55 aa from end of integral membrane protein
31 3917 left=ACAATGACCGAAAAGTTAAG sample=T ref=C right=GTGCAAGACATTATTTTAAC		X	X					X	X	X		Contig 32	50:50 in ref, 8 reads, probably not real
32 16002 left=TTGTTCATTTGACATGATTC sample=A ref=T right=AGCAACTTATTTAATTTTAA	X	X	X	X	X	X	X	X	X	X	X	Contig 33	50:50 in ref, 2 reads, probably not real
35 7083 left=TACGCCAACGAACGATTCGG sample=T ref=C right=TGAAGCAAATGCGATTACAA			X									Contig 36	GCT-GTT=A-V in oxoacyl-ACP synthase
36 166 left=AATTCAGGAATCAAATCTAG sample=C ref=T right=TCATTTGGATGTACGGTTTC			X									Contig 37	GAA-GAG=E-E silent mutation in aspartate kinase
36 10906 left=TAATCTTTTTTAATATTTAT sample=T ref=C right=ACATAAGTTCATTCACTTAA	X	X	X	X	X	X		X		X		Contig 37	Low coverage, no gene predicted by Glimmer
41 4873 left=CGTCGTTATCTACGAATGGT sample=G ref=A right=GGGTATCTAGGTAATCAATG					X	X	X					Contig 42	CTA-CCA=L-P in putative dienelactone hydrolase (no gene predicted by Glimmer)
44 4318 left=TGCAATCTTCAAAGTAGGGG sample=C ref=T right=GTAAAATGTACTTTGGTTAA		X	X		X	X	X	X	X	X	X	Contig 45	Only one read in this region of contig, most likely no differences
46 2472 left=GATAGCCATCCCTTTTTTTT sample= ref=T right=ACGGTTTTCACGTAAAAACG												Contig 47	Intergenic region according to Glimmer, maybe no difference
48 3870 left=TTTGCCATCTTTGTTCCTCC sample=G ref=A right=GTTGTCATTTTTAAATTTCA					X	X	X					Contig 49	Trigger factor. Silent mutation. Only three reads, end of one of them, but also discovered in PacBio genome
55 1802 left=CGTAGTTTTGCAAATGAATC sample=A ref=T right=AGAGGAACACTTTTTCAAAG	X	X	X		X			X	X	X	X	Contig 56	Not a real difference
58 5397 left=AAAGTTTGACAAAAAAATAG sample=C ref=A right=AACCTGCTTATAATACAGAT	X	X	X	X	X	X		X		X	X	Contig 59	Only one read in ref, low qual, not a real difference

VAAL report (contig, position in contig, sequence context)	WT	JK1	JK2	JK3	JK4	JK5	JK6	JK8	JK9	JK10	JK11	Contig No.	Comment
63 3733 left=GTAATTGTTACCGGGATGAG sample=C ref=T right=GGTGCCGGTAAAACAGTTGC					X							Contig 64	AGT-AGC=silent mutation in nucleotide-binding protein
68 65 left=TAAAGAAATTCACAATATAT sample=A ref=G right=TTTTTAAGGAGCTCTTATTA		X	X		X	X	X	X		X		Contig 69	Only one read in this region of contig, most likely no differences. 20 nt upstream of gene predicted by Glimmer
68 1282 left=GTACGACTTATCGGCAAAAC sample=T ref=C right=GACATTCTCCGAAACAAGAT		X										Contig 69	GGT-AGT=G-S in gene with similarity to HTH AraC regulatory protein
72 2466 left=CCCAGAACTTGGGTGGGGGG sample=G ref= right=ATGATGCAAAAGTTAACCAC		X										Contig 73	Frameshift after amino acid 187 in PTS system mannose family transporter subunit IID protein. Verified by looking at the reads
76 232 left=GAGGTTGCCAAGGCGCATCA sample=T ref=C right=GTTGATGCAATTCATCCAGG					X	X	X					Contig 77	CAC-CAT=Silent mutation in pyruvate carboxylase
78 2181 left=TTTGAGCAAATGTTGCCAGT sample=T ref=C right=ATCCCCGTTAACTTGCGCAT		X										Contig 79	GAC-AAC=D-N in putative uncharaterized protein
81 10848 left=AAACAATGATAAGTATATGT sample=G ref=T right=GCTTGTCGTTTTGTCTGCTT	X		X		X	X		X	X	X		Contig 82	Only one read in ref, low qual, not a real difference
81 10861 left=TATATGTTGCTTGTCGTTTT sample=T ref=G right=TCTGCTTTGAACTTTGCTGT	X		X		X	X		X	X	X		Contig 82	Only one read in ref, low qual, not a real difference
83 4262 left=TAAAGTCATTAAAAGTAAAG sample=C ref=T right=ATTTACTCAATTTTGAGTAA											X	Contig 84	BLAST and Glimmer: not coding region, downstream of GTP-binding protein TypA gene
84 6127 left=TACACAAATACAGTGGTACA sample=T ref=C right=TAATAAAAATATGGACTTAT	X	X	X	X	X	X	X	X	X	X	X	Contig 85	Only two reads in contig, one T, one C. Not a real difference
84 8116 left=ATAAGCACCAGCAACAAAAA sample=A ref= right=GCAAAGCGTGCGGCAAAGAA											X	Contig 85	AAA-AAAA=frame shift towards end of Glimmer prediction, no Blast similarity to anything
86 379 left=GTCCTCTTTATTATTTTGGG sample=C ref=T right=TTGGGGGTAATTATATTTGG	X	X	X	X	X	X	X	X	X	X	X	Contig 87	Only one read in ref, low qual, not a real difference
100 3153 left=AAAAAATGTAGAAAGGAGTG sample=C ref=A right=GCCATGTACTTTGATGTAAG		X	X	X	X	X	X	X		X	X	Contig 101	Only 2 reads, M in consensus, not a real difference
100 6822 left=GGTTGGAGCAGTATTTGCTT sample=T ref=C right=GTTGAAAACTTTGAGATCTA		X	X	X	X	X	X	X	X	X	X	Contig 101	Only 2 reads, Y in consensus, not a real difference
102 927 left=CGCAATCCCGTAGCAATCAC sample=G ref=A right=ATGTTTTCAGCAGTGATGGG										X		Contig 103	ATT-ATC=silent mutation in glutathion reductase
110 1947 left=AAATTAAATGGCTTTAAAAA sample=C ref=T right=GCTTATGGAACAGTCTTGTT						X						Contig 111	AAT-AAC=silent mutation in oxidoreductase
113 6644 left=GACAGGCAAACTATTAGGTG sample=A ref=G right=ATGTATTGAAAGTTTGTTTG							X					Contig 114	Only 2 reads in consensus. GGA-GAA=G-E in MccC family protein - putative peptidase
131 1555 left=ATCCACATTTGAGTTTTTTT sample= ref=T right=GTCAAAATGTTGTTACGTTG											X	Contig 132	Intergenic region
136 3247 left=TATATAAATAAAACGAGTCA sample=T ref=G right=GGAACTGAGGTACCTGCATG		X	X	X	X	X	X	X	X	X	X	Contig 137	only one read in consensus. No mutation

VAAL report (contig, position in contig, sequence context)	WT	JK1	JK2	JK3	JK4	JK5	JK6	JK8	JK9	JK10	JK11	Contig No.	Comment
136 3265 left=CAGGGAACTGAGGTACCTGC sample=T ref=A right=TGGAAAGGCGCTCCACAGCC		X	X	X	X	X	X	X	X	X	X	Contig 137	only one read in consensus. No mutation
145 970 left=ACTTATTCAATTGATTGAGC sample=G ref=A right=GGTTGCCAAGAAACATAACC						X						Contig 146	CAG-CGG=Q-R in acetylornithine deacetylase
152 1130 left=TTAATTAAACCATAAAAAAA sample= ref=A right=GAACACGACTTTACGGTCGC			X									Contig 153	6 nt downstream of penicillin-binding protein/beta-lactamase
159 2010 left=GTATGCCACCTCAGGGGGGG sample=G ref= right=TTATTTTATAGAGATTCAAT										X		Contig 160	6 nt downstream of conserved hypothetical protein, putative receptor
195 1078 left=AGATTAACATATTTTGATAA sample=A ref= right=CATAAACTAGGGCGACTAAT	X	X	X		X	X	X	X	X	X	X	Contig 196	Only one read in consensus, most likely no mutation
220 497 left=AATGCCTTATCTCATAGTCT sample=T ref=A right=GAAGCTGCTCGCTGAGCCTT		X	X	X	X	X	X	X	X	X	X	Contig 221	only 2 reads in contig, ambiguity in this position, no mutation
226 422 left=ATAAAGACCTAACCGTCCTG sample=G ref=T right=CACTAAATGCTTATAAAAAT	X	X	X	X	X	X	X	X	X	X	X	Contig 227	Only 1 read in contig, low qual. in this position, no mutation
